# Improving suicide prevention in men

**DOI:** 10.1371/journal.pmed.1005169

**Published:** 2026-06-30

**Authors:** Seena Fazel

**Affiliations:** 1 Department of Psychiatry, University of Oxford, Oxford, United Kingdom; 2 Oxford Health NHS Foundation Trust, Oxford, United Kingdom

## Abstract

Despite public health strategies focusing on men’s mental health and suicide risk, rates of suicide among men remain concerningly high. In this Editorial, Seena Fazel outlines why targeted, specialized approaches for high-risk individuals should be prioritized alongside broader population-level strategies.

## Teaser

Despite public health strategies focusing on men’s mental health and suicide risk, rates of suicide among men remain concerningly high. Targeted, specialized approaches for high-risk individuals should be prioritized alongside broader population-level strategies.

It is widely known that suicide risk disproportionately affects men, who are three times more likely to die from suicide than women and account for 75%–80% of suicide deaths in high-income countries [1]. Globally, there were an estimated 746,000 deaths in 2021 and the mean age of death was 47 years. Public health strategies have increasingly focused on population-level approaches, including reducing stigma, improving mental health literacy, promoting help-seeking, and addressing social determinants of health. These are important developments—with some observational evidence in support [2]—that have broadened understanding of suicide beyond purely clinical models. However, there is a risk that an increasing emphasis on universal prevention can obscure the need for evidence-led targeted interventions. Many of the men at greatest risk of suicide are already identifiable to services. They frequently present with complex combinations of psychiatric disorders, substance misuse, self-harm, physical illness, or criminal justice involvement [3]. Yet many continue to experience fragmented care, poor continuity of support, and limited access to the intensive interventions their needs require. The challenge for suicide prevention is therefore not simply to reduce risk across the population but also to improve outcomes for a smaller but high-risk group of men in whom multiple risk factors accumulate.

Some key contributors to suicide risk remain relatively underemphasized within prevention strategies. Alcohol misuse is one of the strongest and most consistent risk factors for suicidal behaviour among men [4]. Alcohol contributes to increased suicide risk through multiple pathways, including increased impulsivity, impaired judgement, depression, relationship breakdown, unemployment, and social exclusion. Yet alcohol-related harm is often treated as a separate public health issue rather than as a core component of suicide prevention. Similarly, gambling-related harm has gained increasing recognition as an important public health concern [5]. Gambling-related harm can contribute to suicide risk through financial difficulties, debt, relationship conflict, shame, and co-occurring mental health problems. Despite growing evidence linking gambling-related harm to suicide, gambling remains relatively peripheral to many suicide prevention frameworks internationally, although England’s Suicide Prevention Strategy 2023-2028 represents an important step in this direction. Given that men experience elevated levels of both harmful gambling and suicide mortality, integrating gambling-related harms more fully into suicide prevention frameworks, including through support, treatment and prevention services, warrants greater attention and research.

Furthermore, alcohol misuse, gambling problems, psychiatric disorders, and suicidal behavior can cluster within the same individuals. This highlights a broader limitation of some approaches to prevention. Risk factors are often considered individually, while less attention is paid to populations in whom multiple risks converge.

High risk groups include working age adults and older adult men with a history of self-harm, who remain among those at highest risk of subsequent suicide [6]. Systematic reviews have estimated that around 80% of individuals that died from suicide had contact with healthcare services in the year prior, highlighting possible opportunities for intervention. Likewise, some men present to primary care with psychological distress, substance misuse, sleep disturbance, and chronic pain, which are associated with elevated suicide risk. However, access to specialist mental health services can be complicated because they do not meet service thresholds, disengage during referral processes, or fall between organizational boundaries.

Men who move repeatedly through the criminal justice system represent another high risk population [7] and are highlighted in the England’s current National Suicide Prevention Strategy. Substance misuse, psychiatric disorders, homelessness, and gambling-related harms are common in this group. Contact with police, courts, prisons, and probation services provide another opportunity to identify elevated risk, and in younger men. However, care pathways are often fragmented and continuity of care can be poor. Responsibility for individuals with complex needs is often dispersed across multiple agencies without effective coordination.

The problem, therefore, extends beyond risk identification to access and links with services and appropriately targeted treatments. How current service models meet this challenge is uncertain. Recent years have seen growing interest in transdiagnostic and symptom-focused approaches to mental healthcare—approaches which provide information about prognosis, likely treatment response, patterns of risk, and appropriate interventions [8]. While these developments have many strengths, the relevance of diagnosis has been undervalued.

Across many healthcare systems there has been a shift towards streamlined assessment pathways, standardized interventions, and generic models of care. One example is the UK’s Improving Access to Psychological Therapies programme, which is an NHS primary care mental health service that provides standardized psychological interventions, predominantly for common mental health disorders. Such approaches may improve throughput and increase access for some patients with common mental disorders, such as anxiety and those with depressive symptoms and stress-related problems. However, they can be less effective for individuals whose difficulties span multiple domains, and those with more serious mental health needs. Meaningful assessment of suicide risk in these circumstances is unlikely to be straightforward.

For high-risk men, diagnostic clarity can be particularly important. Alcohol dependence, depressive disorders, bipolar disorder, personality disorders, post-traumatic stress disorder, and neurodevelopmental conditions require different treatments for underlying conditions and comorbidities [9]. Moreover, establishing rapport with individuals who have experienced repeated service failures, distrust institutions, or struggle to articulate emotional distress often requires time, continuity, and clinical expertise. This may be particularly the case for older men, who can present with social withdrawal, alcohol misuse, physical illness, or behavioral change, and often minimize their problems, rather than explicitly reporting suicidal thoughts. Early intervention therefore remains important, and some men require assertive outreach, specialist addiction treatment, intensive psychological therapies, integrated care models, and sustained multidisciplinary support. However, these services are resource-intensive, and research examining this has been mostly conducted in high-income countries. The role of these service models in low- and middle-income countries, where the availability of specialist staff and resources is typically much more constrained, is less clear; here, digital interventions could improve access [10]. Improvements in digital infrastructure could also assist with flagging risks and needs in people who are involved with different services, and a clearer understanding of principles underlying confidentiality that informs sharing information about risks. In most jurisdictions, confidentiality is an important legal and ethical duty but healthcare professionals can disclose personal information justified in the public interest, which includes risk to the individual of serious harm.

Future progress in suicide prevention will depend on maintaining a balance between population-level prevention and targeted intervention for those at highest risk. Alcohol misuse, gambling-related harm, self-harm, psychiatric disorders, and criminal justice involvement should be more explicitly integrated into prevention strategies. Equally important is strengthening pathways between primary care, specialist mental health services, addiction services, and criminal justice agencies. Many men who die by suicide are those who repeatedly encounter healthcare services without receiving sustained, coordinated, and personalized care. Reducing suicide among men will require more than awareness campaigns and generalized interventions. It will require investment in specialist expertise, improved risk assessment informed by contemporary evidence on risk factors and scalable assessment tools [11], diagnostic precision, continuity of care, and services that can engage people whose needs are complex.
